# Development and validation of an interpretable machine learning model for predicting in-hospital hypoglycemia in adults with type 1 diabetes mellitus: a multicenter retrospective study

**DOI:** 10.3389/fendo.2026.1816599

**Published:** 2026-04-17

**Authors:** Qiang Zhang, Salama Habibu Saad, Haojie Zhou, Bianca Tankie Baakile, Yanfen Fu, Ran Li, Ruolin Han, Qian Tang, Lingyan Xuan, Guoyan He, Hui Li, Yuan Zhao, Niqing Zhu, Jianjun Zou, Xiaoli Zhu

**Affiliations:** 1 School of Nursing, Dali University, Yunnan, China; 2 School of Basic Medicine and Clinical Pharmacy, China Pharmaceutical University, Jiangsu, China; 3 Department of Pharmacy, Nanjing First Hospital, Nanjing Medical University, Jiangsu, China; 4 Department of Cardiovascular Medicine, The Second Affiliated Hospital of Chongqing Medical University, Chongqing, China; 5 Department of Endocrinology, First Affiliated Hospital of Kunming Medical University, Yunnan, China; 6 Department of Endocrinology, The First Affiliated Hospital of Dali University, Yunnan, China; 7 Department of Nursing, Second People’s Hospital of Qujing City, Yunnan, China; 8 Department of Endocrinology and Metabolism, Pu’er People’s Hospital, Yunnan, China; 9 School of Public Health, Dali University, Yunnan, China; 10 First Clinical Medical College, Southern Medical University, Guangdong, China

**Keywords:** hypoglycemia, machine learning, multicenter, predictive model, type 1 diabetes mellitus

## Abstract

**Background:**

In-hospital hypoglycemia remains a serious and potentially life-threatening complication among adults with type 1 diabetes mellitus (T1DM), yet reliable and interpretable prediction tools for Chinese inpatients are lacking. We aimed to develop and validate an interpretable machine learning model using multicenter inpatient data to predict the risk of in-hospital hypoglycemia in adults with T1DM, and to enhance clinical understanding of key predictors.

**Methods:**

This multicenter retrospective cohort study enrolled adult inpatients with T1DM from five tertiary Grade A hospitals in China between January 1, 2019 and September 30, 2025. From the same multicenter cohort, the total dataset was randomly split 7:3 into a development set (*n* = 1,048) and an independent external validation set (*n* = 450). Within the development set, we performed 5-fold stratified cross-validation for hyperparameter tuning, and both internal cross-validation and external validation remained fully independent throughout model development. Machine learning models were trained to predict in-hospital hypoglycemia and evaluated for discrimination, calibration, clinical utility, and interpretability.

**Results:**

The study enrolled 1,498 patients, of whom 580 (38.7%) experienced in-hospital hypoglycemia. The random forest model demonstrated superior predictive performance in the external validation cohort, achieving an AUC of 0.831 (*95% CI*: 0.798-0.873), sensitivity of 0.793, specificity of 0.748, and a Brier score of 0.149. Hemoglobin, potassium, sodium, low-density lipoprotein cholesterol, and age at onset were identified as the top predictors. Hemoglobin, potassium, sodium, and BMI exhibited U-shaped associations with hypoglycemia risk, where both low and high values increased risk. Exploratory analysis of joint biomarker status showed that patients with abnormalities in two or more of these core predictors had a non-significant trend toward higher event rates, while the complexity of their combined effects was better captured by the non-linear model. The model enabled effective risk stratification into four quartiles, and decision curve analysis confirmed its consistent net clinical benefit across relevant probability thresholds.

**Conclusions:**

The interpretable random forest model using routine inpatient data showed strong discrimination, good calibration and useful risk stratification for in-hospital hypoglycemia in Chinese adults with T1DM, which may help identify high-risk patients early and guide targeted preventive interventions in clinical practice.

## 1 Introduction

Type 1 Diabetes Mellitus (T1DM) features irreversible pancreatic β-cell dysfunction, requiring lifelong exogenous insulin to maintain euglycemia (1). Hypoglycemia remains a major clinical challenge and threatens the safety of hospitalized patients with this condition, who face higher risk than ambulatory counterparts due to multimorbidity, frequent regimen adjustments and stress responses disrupting glycemic balance (2). A multicenter study of 8,022 insulin-treated patients with T1DM reported 73.3 annual hypoglycemia episodes per patient-year, with 0.24 being severe and requiring hospitalization (3). This highlights the global clinical burden of the complication. Danish data confirmed prior hypoglycemia-related hospitalization as the strongest predictor of recurrence, with risk rising in a dose-dependent way (4).

In hospitalized patients with T1DM, the acute and heterogeneous nature of glycemic fluctuations complicates early detection of hypoglycemia. Some patients develop asymptomatic hypoglycemia that may be missed by routine point-of-care glucose monitoring, especially when continuous glucose monitoring is not universally available. Hypoglycemia in hospitalized Chinese patients with T1DM exhibits population-specific characteristics related to incidence, hypoglycemia unawareness, baseline control, and associated adverse outcomes, reflecting differences in genetic background, diet, and clinical management (5, 6). These events increase the risks of cognitive decline, major adverse cardiovascular events, prolonged hospital stays, and higher healthcare utilization (7, 8).

Machine learning methods are well suited to hypoglycemia prediction because they can integrate multidimensional predictors and capture nonlinear relationships that traditional regression models may overlook (9). However, three key limitations hinder the clinical translation of existing hypoglycemia prediction models. First, Chinese adult-onset autoimmune diabetes differs from Western populations in insulin sensitivity, body composition, and dietary patterns, limiting the transportability of existing models developed primarily in Western cohorts (6, 10). Second, inpatient-specific variables such as acute comorbidities, in-hospital insulin regimens, and time-varying glycemic variability metrics are often underused in model construction, despite their close association with glycemic fluctuations (11). Third, many models lack explicit interpretability tools to quantify the contribution of individual predictors, reducing clinician confidence and impeding clinical implementation (12). Therefore, developing an interpretable hypoglycemia risk prediction model based on multicenter inpatient data from the Chinese T1DM population has become an urgent clinical need to improve patient prognosis.

To address these gaps, we conducted a multicenter retrospective study in Chinese adult T1DM inpatients to develop and validate a machine learning-based prediction model for in-hospital hypoglycemia. We used routinely collected electronic health record data, systematically compared 4 supervised algorithms, selected the best model based on predefined performance criteria, and applied SHAP and restricted cubic spline analyses to provide transparent, clinically meaningful interpretation. We also evaluated clinical utility using decision curve analysis and constructed risk stratification schemes to facilitate bedside use. This study aims to provide a reliable tool for early high-risk identification, reducing inpatient hypoglycemia and adverse outcomes.

## 2 Materials and methods

### 2.1 Study design, participants and data source

This retrospective multicenter cohort study used electronic health records from 5 tertiary grade A hospitals in China. Adult inpatients (aged ≥ 18 years) with a primary diagnosis of T1DM between January 1, 2019, and September 30, 2025, were eligible for inclusion. T1DM diagnoses were confirmed by manual review of International Classification of Diseases, Ninth and Tenth Revision (ICD-9/ICD-10) codes and clinical records. Exclusion criteria were (1) admission primarily for hypoglycemia (2); cognitive impairment or documented psychiatric disorders.

The study was approved by the ethics committees of all participating centers, with informed consent waived for its retrospective design, and adhered to TRIPOD + AI statement for prediction model development and reporting (13).

### 2.2 Predictors, outcome, and data extraction

Potential predictors were identified through a systematic literature review of hypoglycemia risk factors in hospitalized T1DM patients (14) and refined via 2-round Delphi consensus among 15 endocrinologists. Sixty-five clinically plausible variables were retained and grouped into 4 categories: (1) demographic characteristics; (2) diabetes-related clinical factors; (3) comorbidities; (4) laboratory parameters.

The primary outcome was defined as the occurrence of in-hospital hypoglycemia, defined as a capillary blood glucose level < 3.9 mmol/L documented in the EMR system (15). Patients were divided based on whether they had in-hospital hypoglycemia.

Data were extracted from hospital electronic medical records. The last measurement before discharge was collected for patients without in-hospital hypoglycemia and the last measurement preceding the episode for those with hypoglycemia.

### 2.3 Missing data handling

Each predictor’s missingness has been determined as the total percentage of missing values for each encounter based on the number of encounters. Any predictors with greater than 40% missingness from the model have been omitted (16). The remaining predictors were handled through a standardized preprocessing pipeline using median imputation and z-score standardization for numeric predictors, and mode imputation and one-hot encoding for categorical predictors with the first category dropped and unknown levels excluded (17, 18). The above pre-processing steps were performed to ensure the absence of missing or infinite values in the analytic dataset and consistency in the number of observations between the imputation dataset and the original outcome data.

### 2.4 Model development, validation, performance evaluation and interpretation

Eligible encounters were randomly divided into a development cohort (70%) and an external validation cohort (30%) via stratified random sampling based on hypoglycemia status, ensuring consistent event rates between the two cohorts. The development cohort was used for feature preprocessing, model training, internal validation, and hyperparameter tuning, whereas the external validation cohort was held out and used only for final performance evaluation and clinical utility analyses.

Four supervised machine learning algorithms were employed, including random forest (RF), eXtreme Gradient Boosting (XGBoost), decision tree (DT), and logistic regression (LR). Within the development cohort, we used 5-fold stratified cross-validation to optimize hyperparameters with AUC as the primary scoring metric. After tuning, we refit each algorithm on the full development cohort using the selected hyperparameters and the same preprocessing pipeline. The fitted preprocessing pipeline and model parameters were then applied to the external validation cohort without refitting or re-tuning on external data to avoid information leakage.

### 2.5 Performance assessment and threshold selection

The primary assessment of model performance was conducted in the external validation cohort to quantify out-of-sample predictive validity. Model discrimination was evaluated using AUC with 95% confidence intervals obtained from 1000 bootstrap resamples. Calibration was assessed by Brier score and calibration curves comparing predicted probabilities with observed frequencies across deciles of predicted risk. To characterize model performance under class imbalance, we examined precision recall curves and average precision. In the development cohort (internal validation), a predefined operating threshold will be selected using the Youden’s J statistic, based on which the accuracy, sensitivity, specificity, precision, and F1-score of each model will be calculated. Four machine learning algorithms will be applied to derive the optimal probability threshold for generating these performance metrics.

Clinical utility was evaluated via decision curve analysis, comparing the net benefit of using the model to “treat all” and “treat none” strategies across risk thresholds ranging from 0.10 to 0.50. Clinical risk stratification was performed by applying the final RF model to the external validation cohort, defining four risk groups based on predicted probability ranges. The number needed to evaluate (NNE) was calculated within each group, defined as the average number of patients that need to be screened using the model to identify one additional case of hypoglycemia; this is conceptually analogous to the number needed to treat (NNT) but applied to a risk prediction and screening context. Observed hypoglycemia rates were also computed for each risk stratum.

All prespecified primary performance metrics (AUC, sensitivity, specificity, accuracy, F1-score, Brier score), ROC and precision recall curves, calibration plots, decision curves, and risk stratification results were derived from the external validation cohort. Internal cross-validation metrics from the development cohort were used to assess model stability during tuning but were not used for the main clinical interpretation.

### 2.6 Interpretability analyses

For the best-performing algorithm, we computed SHAP values to quantify the contribution of each predictor to individual risk predictions and to derive global feature importance rankings. SHAP summary plots and dependence plots for the top predictors were generated to visualize nonlinear patterns, including U-shaped relationships, and to explore potential interactions. We further applied restricted cubic spline (RCS) regression to selected continuous predictors to confirm and quantify nonlinear relationships between predictor values and hypoglycemia risk.

### 2.7 Joint effects of biomarkers and clinical decision rules

Using RCS and SHAP findings, we defined exploratory “normal” ranges for hemoglobin, serum potassium, and sodium, and created indicator variables for biomarker abnormalities. Patients were stratified into categories according to the number and pattern of abnormal biomarkers, and multivariable logistic regression models were used to estimate adjusted odds ratios for hypoglycemia, controlling for age, diabetes duration, prior hypoglycemia, and glucose variability. We evaluated pairwise interaction terms and conducted decision curve analysis to compare net benefit between biomarker rule-based strategies and the RF probability-based strategy.

### 2.8 Statistical analysis

Continuous variables were summarized as means ± SD if approximately normally distributed, and as medians (IQR) if skewed. Categorical variables were summarized as counts and percentages. Group differences between encounters with hypoglycemia and without hypoglycemia used the Student’s t-test for normally distributed continuous variables, Mann-Whitney U-test for non-normally distributed continuous variables, and Chi-square test for categorical variables. All statistical tests were all two-sided with P-values < 0.05. Analyses were conducted using R, version 4.1.2 (R Foundation for Statistical Computing) and Python, version 3.7 (Python Software Foundation) with standard machine learning and SHAP libraries (19, 20).

## 3 Results

### 3.1 Characteristics of patients

**Figure 1:**
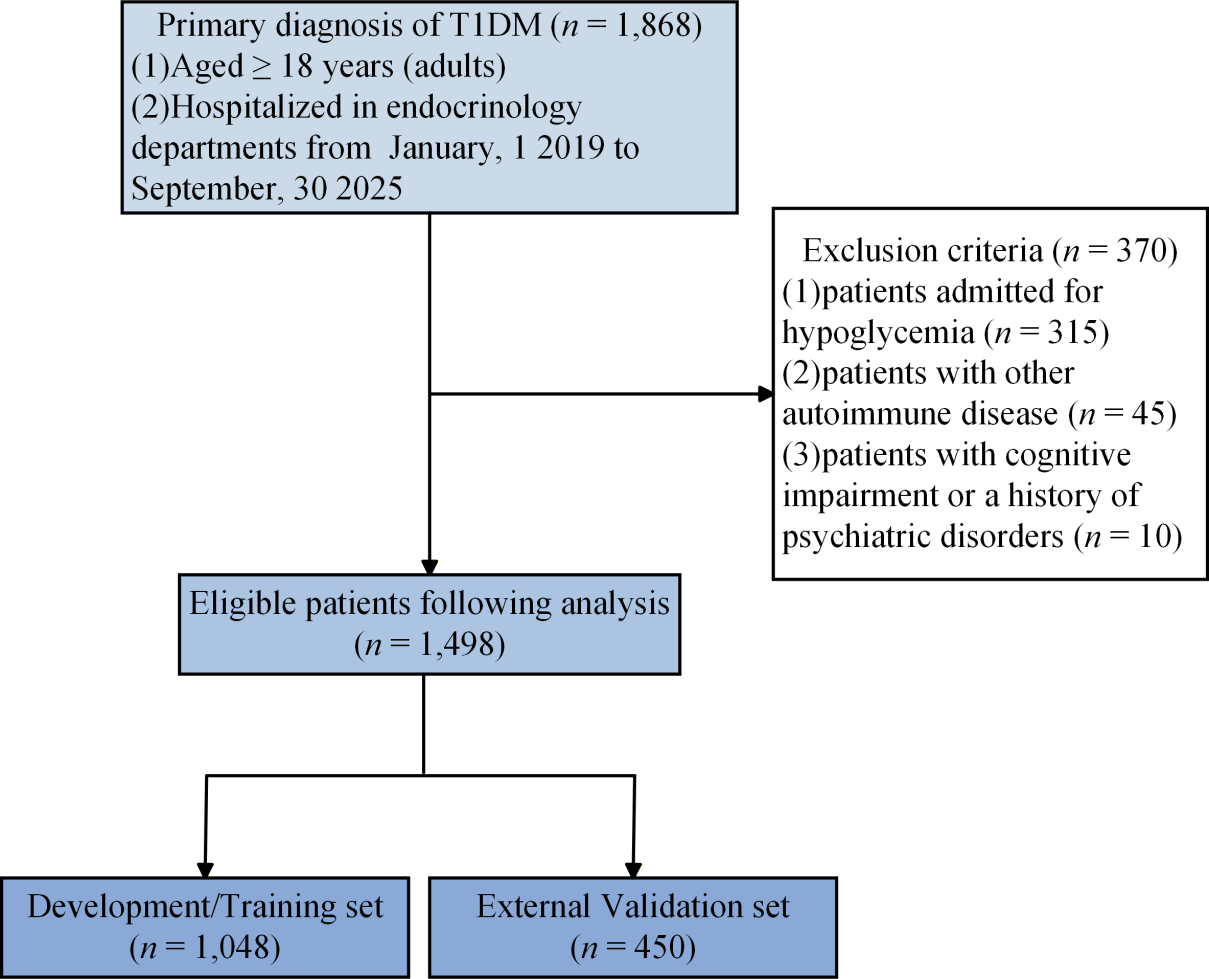
Study cohort flow diagram.

A total of 1,498 adult T1DM inpatients met the inclusion and exclusion criteria and were included in the analysis (**Figure 1**). Following data processing, 12 variables with a missing proportion exceeding 40% were excluded. Among the 65 variables initially collected in this study, 53 were ultimately included for subsequent analysis, and detailed information on all 65 original variables is presented in **Supplementary Table 1**. The mean age of the study population was 42.3 ± 15.7 years, and the mean diabetes duration was 16.2 ± 9.8 years. Overall, 580 patients (38.7%) experienced at least 1 hypoglycemic episode during hospitalization. The development cohort (*n* = 1,048) and the external validation cohort (*n* = 450) had similar baseline demographic, clinical, and laboratory characteristics, including comparable hypoglycemia incidence (*P* = 0.62), supporting the validity of the stratified split (**Table 1**).

**Table 1:** Baseline characteristics of the development and validation sets.

Characteristics	Overall (*n* = 1,498)	Development (*n* = 1,048)	Validation (*n* = 450)	*P* value
Age (years)	42.3 ± 15.7	41.8 ± 15.9	43.5 ± 15.2	0.08
Sex (female)	712 (47.5%)	502 (47.9%)	210 (46.7%)	0.68
BMI (kg/m²)	23.1 ± 4.2	23.0 ± 4.3	23.3 ± 4.0	0.25
Disease duration (years)	16.2 ± 9.8	15.9 ± 9.9	16.8 ± 9.6	0.12
Diabetic neuropathy	428 (28.6%)	302 (28.8%)	126 (28.0%)	0.76
Insulin pump use	189 (12.6%)	128 (12.2%)	61 (13.6%)	0.48
Hemoglobin (g/L)	128 ± 21	127 ± 22	129 ± 19	0.10
Potassium (mmol/L)	4.1 ± 0.6	4.1 ± 0.6	4.1 ± 0.5	0.89
Sodium (mmol/L)	139.2 ± 4.3	139.1 ± 4.4	139.4 ± 4.1	0.27
LDL-C (mg/dL)	98.4 ± 32.7	97.8 ± 33.1	99.8 ± 31.8	0.31
Hypoglycemia events	580 (38.7%)	410 (39.1%)	170 (37.8%)	0.62

Demographic, clinical, and laboratory characteristics of the overall study population and stratified by development and validation cohorts. Continuous variables are presented as mean ± standard deviation; categorical variables as *n* (%). *P* values indicate comparisons between development and validation cohorts using Student’s t-test for continuous variables and Chi-square test for categorical variables. All characteristics were well-balanced between cohorts, supporting the validity of the dataset partitioning.

### 3.2 Comparative performance of machine learning models

**Figure 2:**
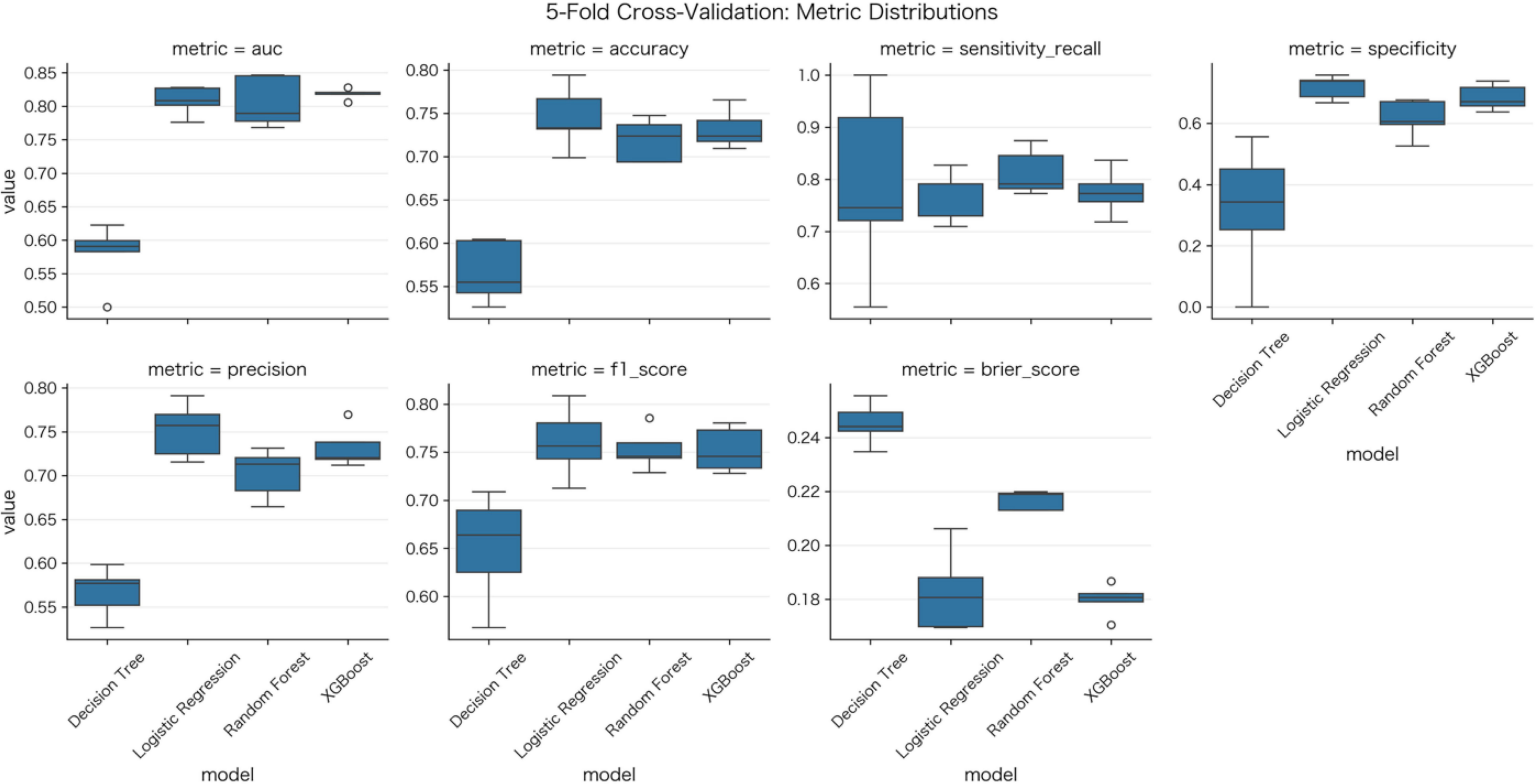
5-Fold cross-validation: metric distributions. Box plots show the distributions of AUC, accuracy, sensitivity/recall, specificity, precision, F1-score, and brier score for decision tree, logistic regression, random forest, and XGBoost models.

**Figure 3:**
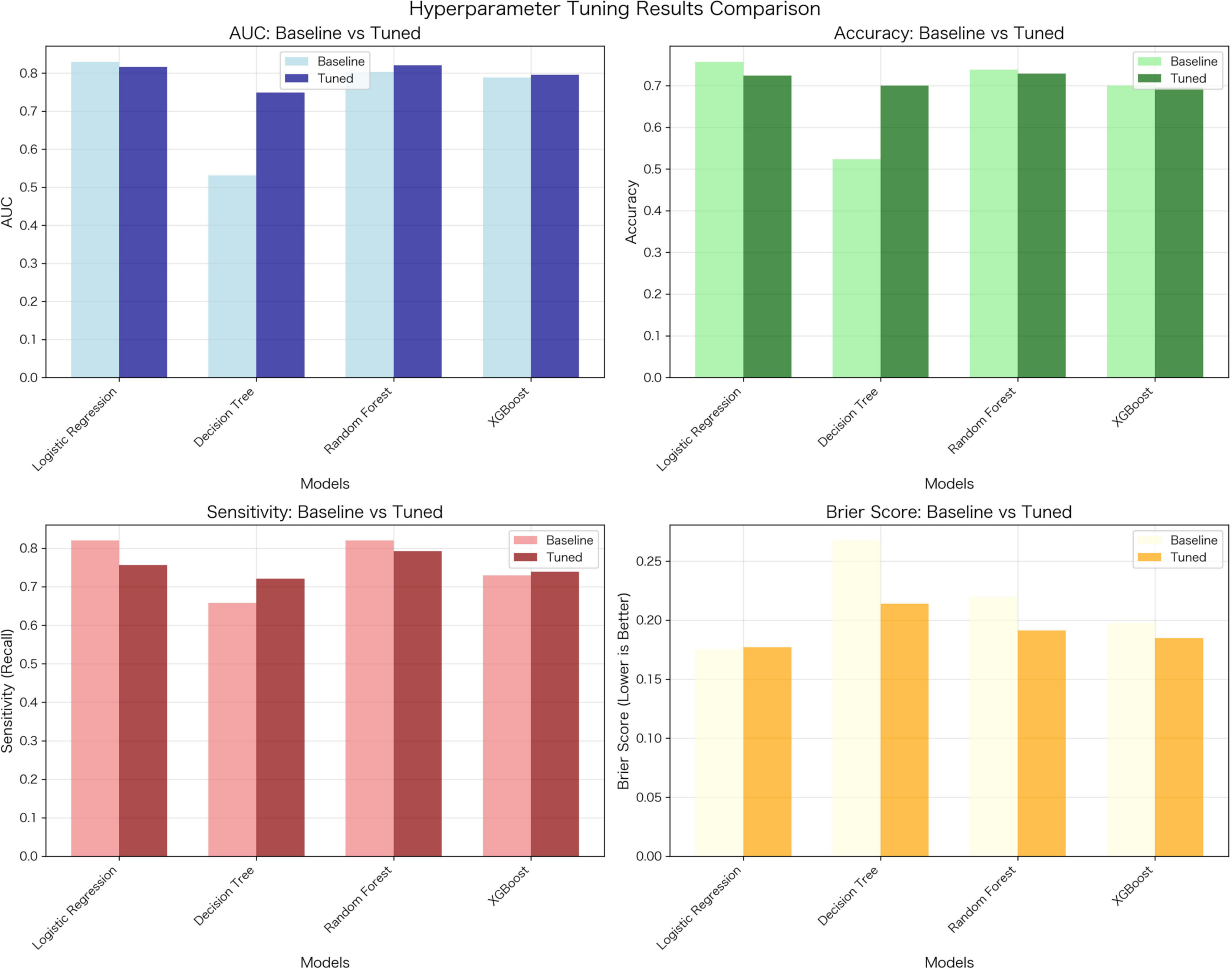
Hyperparameter tuning improves key performance metrics of the hypoglycemia risk prediction model. Bar plots demonstrate significant performance improvements achieved through systematic hyperparameter optimization. Dark bars represent the tuned model performance, while light bars represent the baseline model performance.

During data preprocessing, one-hot encoding was applied to high-cardinality categorical predictors, increasing the number of processed features in the final design matrix to 3,107–3,179. In the development cohort, 5-fold stratified cross-validation was used to tune hyperparameters for all 4 algorithms (**Figure 2**; **Supplementary Text 1**). Following tuning, final models were fit to the full development cohort, and the optimal operating threshold was selected using the Youden’s J statistic based on the internal validation (development) data, which is presented in **Supplementary Table 2**. The final models were then evaluated in the external validation cohort. In external validation, the RF classifier achieved the best overall performance, with an AUC of 0.831 (*95% CI*: 0.789-0.873), sensitivity of 0.793, specificity of 0.748, accuracy of 0.769, F1-score of 0.784, and Brier score of 0.149 (**Figure 3**, **Table 2**). LR yielded an AUC of 0.786 (*95% CI*: 0.741-0.831), XGBoost an AUC of 0.758 (*95% CI*: 0.710-0.806), and DT an AUC of 0.645 (*95% CI*: 0.592-0.698).

**Table 2:** Model performance on external validation.

Model	*AUC* (95% *CI*)	Sensitivity	Specificity	Brier Score	F1 Score
Random Forest (RF)	0.831 (0.789-0.873)	0.793	0.748	0.149	0.784
Logistic Regression (LR)	0.786 (0.741-0.831)	0.735	0.712	0.183	0.752
eXtreme Gradient Boosting (XGBoost)	0.758 (0.710-0.806)	0.718	0.694	0.195	0.731
Decision Tree (DT)	0.645 (0.592-0.698)	0.642	0.628	0.234	0.658

Presents discrimination (*AUC* with 95% *CI*), sensitivity, specificity, calibration (Brier score), and F1-score for four predictive models evaluated on external validation data.

**Figure 4:**
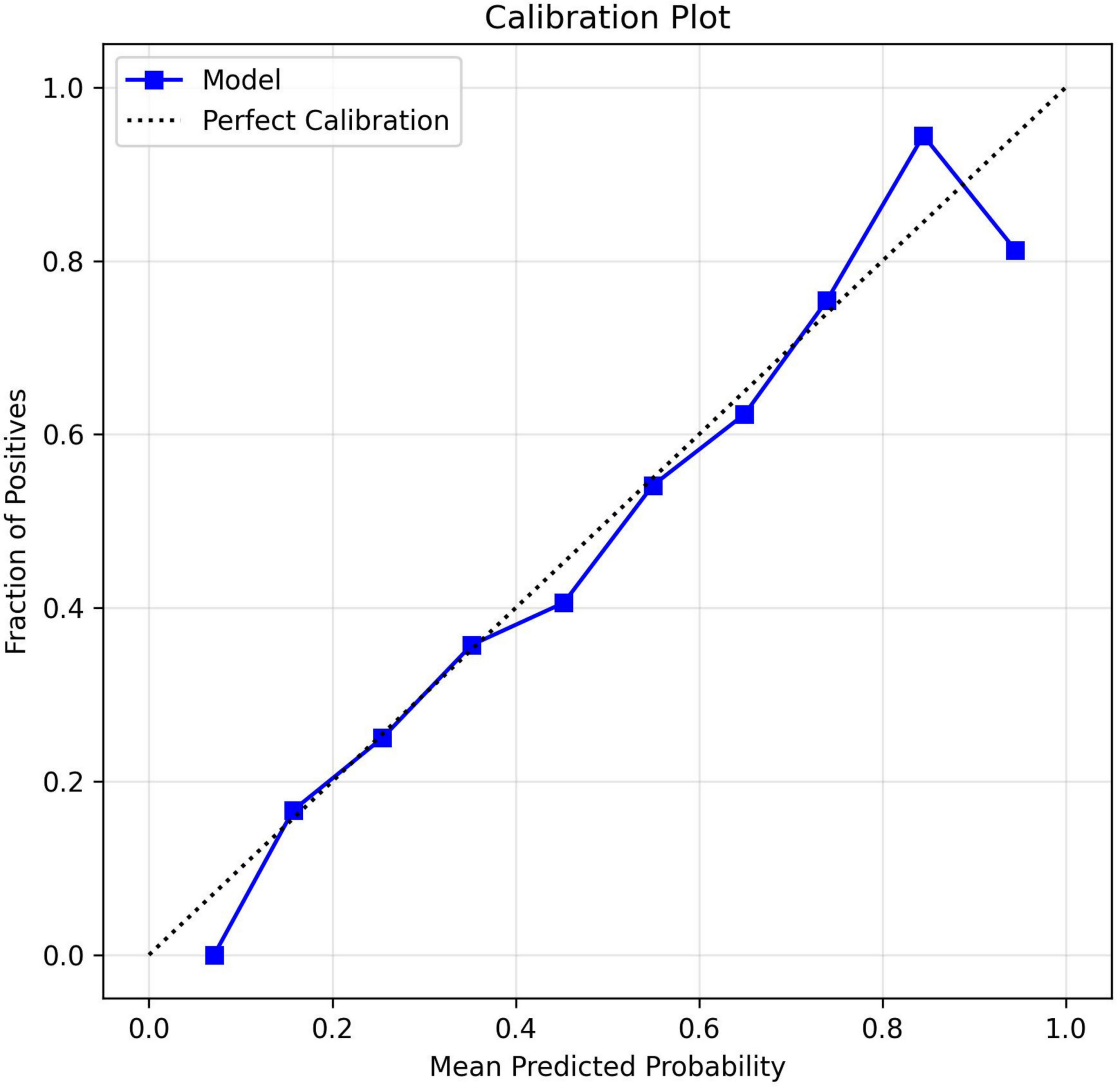
Calibration plot of the random forest model. Calibration curve demonstrating agreement between predicted probabilities and observed hypoglycemia outcomes in external validation cohort. Perfect calibration line (dashed) shown for reference.

Hyperparameter optimization substantially improved RF performance compared with the baseline model, increasing AUC from 0.766 to 0.831 and decreasing the Brier score to 0.149. Calibration plots for the tuned RF classifier in the external validation cohort showed close agreement between predicted and observed probabilities across risk deciles (**Figure 4**).

### 3.3 Precision-recall, calibration, and decision curve analyses

**Figure 5:**
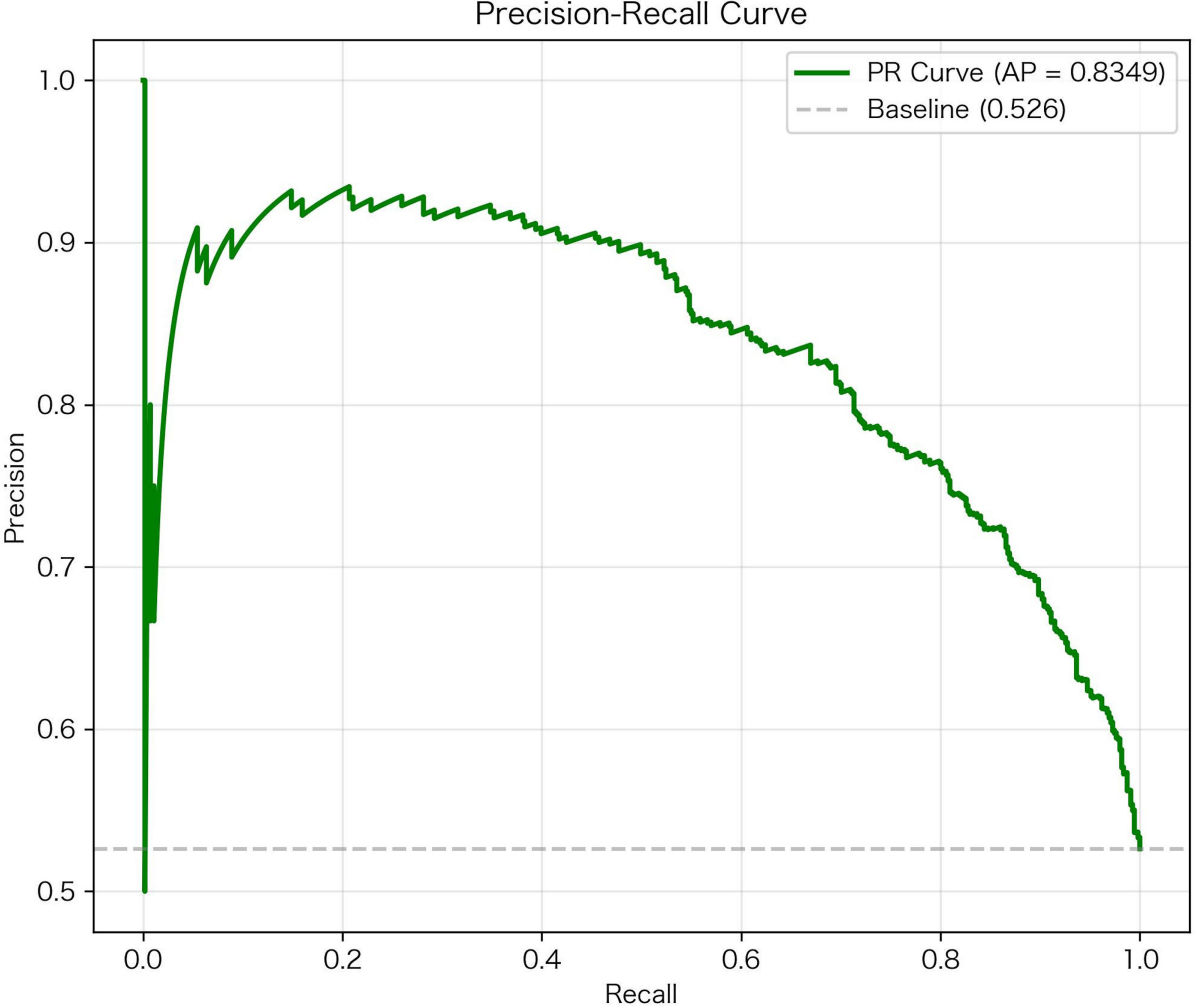
Precision-recall curve of the random forest model for hypoglycemia risk prediction. Precision-recall curve demonstrating the trade-off between precision (positive predictive value) and recall (sensitivity) for the final Random Forest model on external validation.

**Figure 6:**
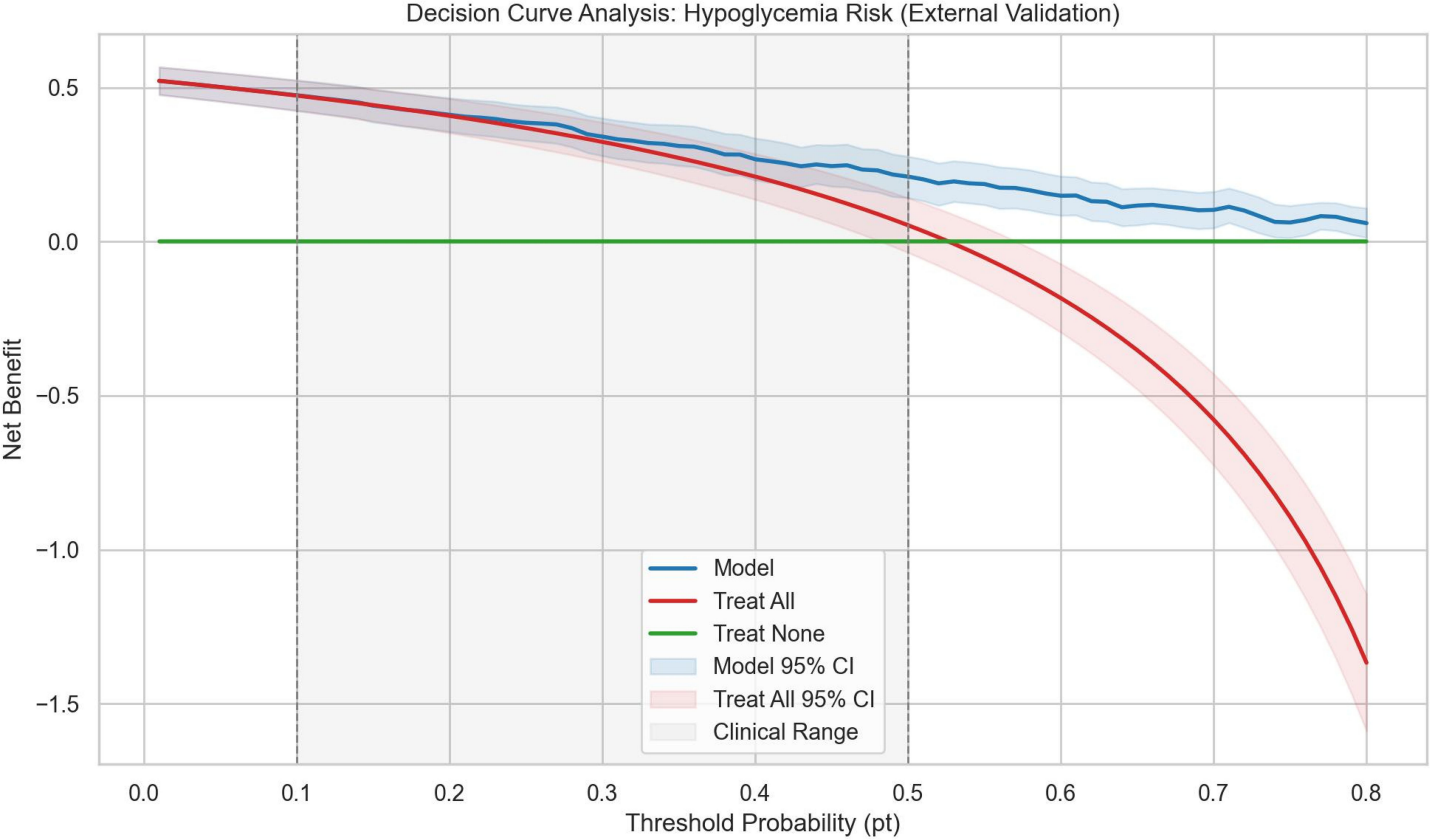
Decision curve analysis of the random forest model for hypoglycemia risk prediction in hospitalized T1DM patients. Decision curve showing the net benefit of using the prediction model across probability thresholds for clinical intervention. The model (blue) demonstrates superior clinical utility compared to ‘treat-all’ and ‘treat-none’ strategies across most clinically relevant thresholds (0.1-0.5), supporting its implementation for targeted hypoglycemia prevention in hospitalized Type 1 diabetes patients.

The RF model received an average of approximately 0.835 in precision on the precision-recall curve in the external validation cohort, with a positive class prevalence of 0.526 (**Figure 5**). This indicates strong performance of the RF model, regardless of the baseline class imbalance. In the calibration analysis, the RF model showed excellent agreement between predicted risk factors and observed frequencies of hypoglycemia based on the low Brier score and by means of the calibration plot. In decision curve analyses, the RF model provided a higher net benefit than either “treat all” or “treat none” models over threshold probabilities from 0.10 to 0.50 (**Figure 6**). Collectively, these results suggest that the RF Model has utility in clinical practice as a guide to implement preventative interventions for individuals at risk of experiencing hypoglycemia.

### 3.4 Clinical risk stratification and event identification efficiency

**Figure 7:**
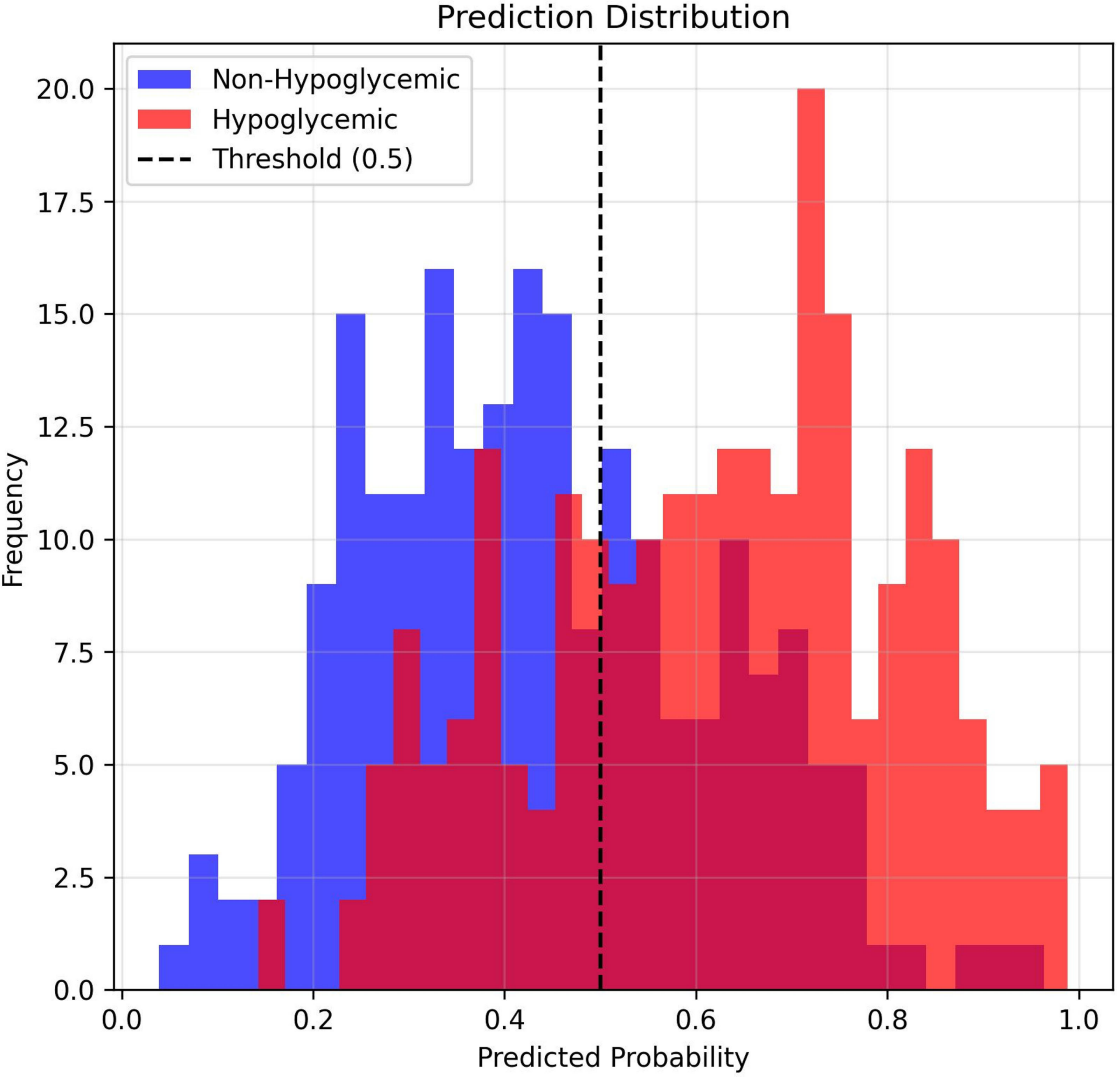
Clinical risk stratification performance Clinical risk stratification using prediction probability thresholds. Vertical lines indicate risk quartile cutoffs (0.23, 0.41, 0.67), enabling categorization of patients into very low (12.3%) to very high (83.5%) risk groups for targeted interventions.

Applying the final RF model to the external validation cohort enabled stratification into 4 risk groups based on predicted hypoglycemia probability: very low (< 0.23), low (0.23-0.41), high (0.42-0.66), and very high (> 0.67). Observed hypoglycemia rates increased across these groups from 12.3% in the very low-risk group to 83.5% in the very high-risk group (**Table 3**, **Figure 7**). The NNE to identify 1 hypoglycemic event decreased from 8.1 in the very low-risk group to 1.2 in the very high-risk group, indicating efficient targeting of high-risk patients.

**Table 3:** Clinical risk stratification in validation.

Risk quartile	Predicted probability Range	Patients, *n*	Hypoglycemia events, *n* (%)	Number needed to evaluate (NNE)
Very Low	< 0.23	113	14 (12.3%)	8.1
Low	0.23–0.41	112	29 (25.9%)	3.9
High	0.42–0.66	112	61 (54.5%)	1.8
Very High	> 0.67	113	94 (83.5%)	1.2
Overall	/	450	198 (44.0%)	2.3

Predicted probability ranges by risk quartile, observed patients, hypoglycemia events, and the number needed to evaluate (NNE) to identify one event.

### 3.5 SHAP-based feature importance and nonlinear relationships

**Figure 8:**
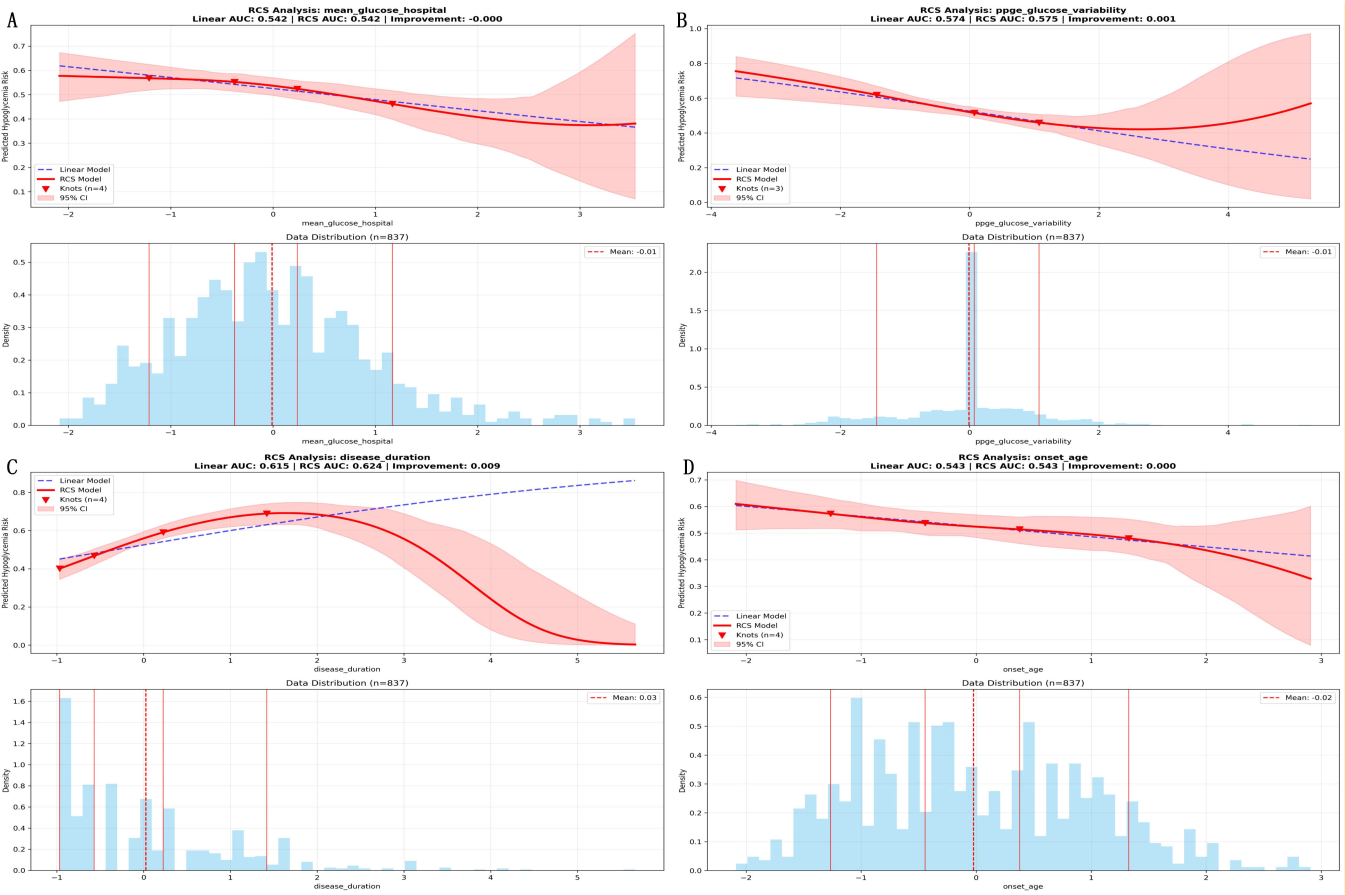
Restricted cubic spline plots illustrating nonlinear relationships between key continuous predictors and hypoglycemia risk. Nonlinear relationships between continuous predictors and hypoglycemia risk. The shaded areas denote clinical normal range: **(A)** mean hospitalization glucose, **(B)** glucose variability, **(C)** disease duration, **(D)** age at diabetes onset.

SHAP analysis of the RF model identified hemoglobin as the most influential predictor of hypoglycemia risk, followed by potassium, sodium, LDL cholesterol, age at onset, postprandial glucose variability, body mass index, diabetes duration, previous hypoglycemia, and mean hospitalization glucose (**Table 4**). SHAP dependence plots for the top 10 predictors (**Supplementary Figure 1**) visually confirmed the U-shaped associations for hemoglobin, potassium, sodium, and body mass index, with the lowest predicted risk observed within physiologic ranges and higher risk at both low and high values. RCS analyses confirmed these nonlinear patterns for electrolytes and identified additional nonlinear associations for mean hospitalization glucose, glucose variability, diabetes duration, and age at onset (**Figure 8**).

**Table 4:** Feature importance from SHAP analysis.

Rank	Feature	Mean SHAP	Direction of effect	Clinical domain
1	Hemoglobin	0.42	U-shaped	Laboratory
2	Potassium	0.38	U-shaped	Laboratory
3	Sodium	0.36	U-shaped	Laboratory
4	LDL-C	0.34	Positive	Laboratory
5	Age at onset	0.31	Negative	Diabetes
6	Postprandial glucose variability	0.28	Positive	Glucose metrics
7	BMI	0.26	U-shaped	Demographic
8	Diabetes duration	0.24	Positive	Diabetes
9	Previous hypoglycemia	0.23	Positive	Clinical
10	Mean glucose hospitalization	0.21	Negative	Glucose metrics

SHAP feature importance ranks with mean SHAP value and direction of effect, grouped by clinical domain.

### 3.6 Biomarker-based risk stratification and clinical event analysis

**Figure 9:**
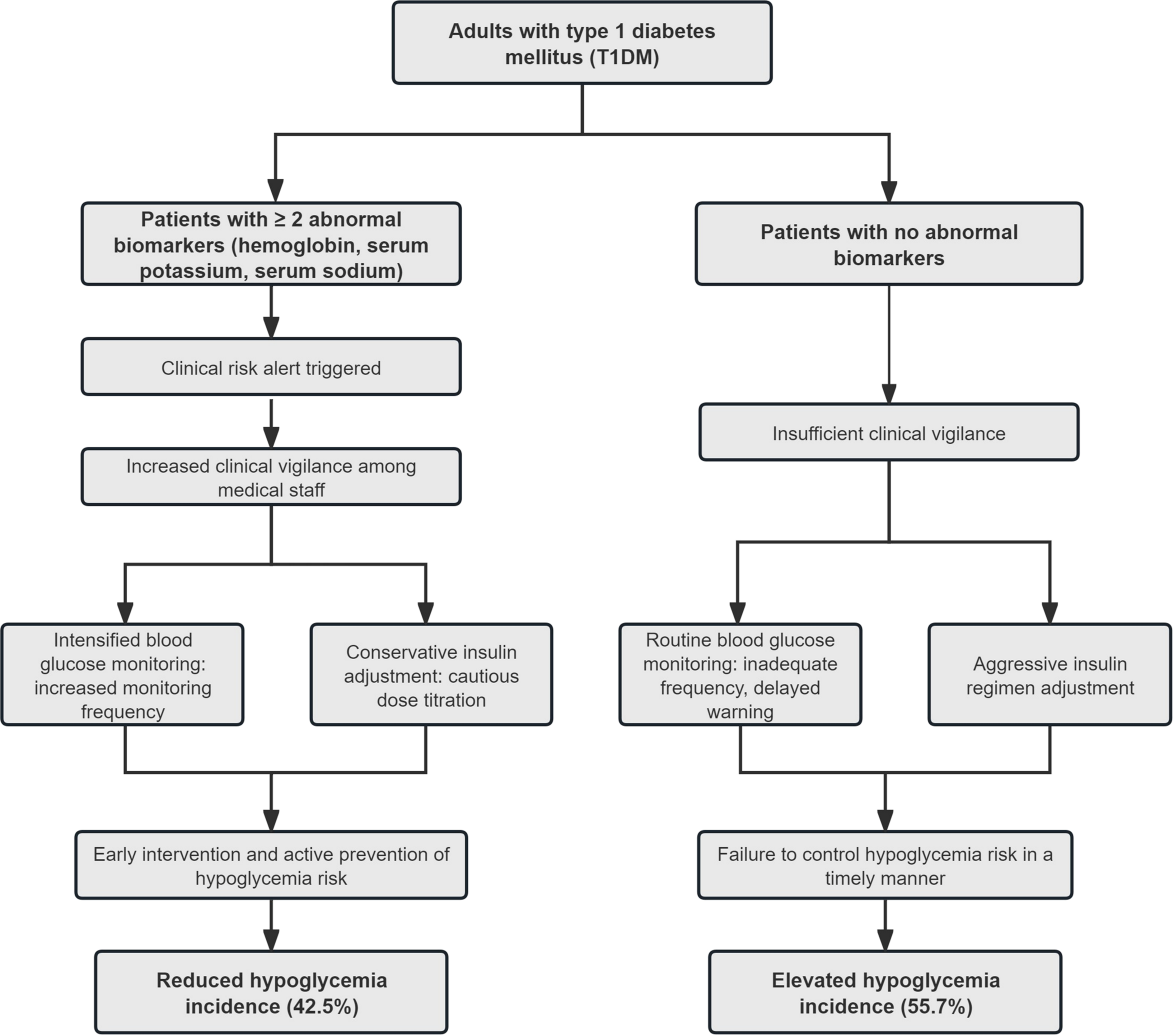
Monitoring bias and hypoglycemia incidence in T1DM. Abnormal biomarkers trigger clinical vigilance and preventive interventions, reducing observed hypoglycemia incidence, while lack of biomarkers leads to insufficient monitoring and higher events, illustrating monitoring bias in clinical practice.

Combining SHAP and RCS findings, exploratory “normal” thresholds were defined for hemoglobin, serum potassium, and sodium **Supplementary Text 2**), and patients were grouped according to the number of abnormal biomarkers (**Table 5**). In clinical event analyses, patients with 2 or more abnormal biomarkers had a hypoglycemia incidence of 42.5%, whereas those with no abnormal biomarkers had the highest incidence (55.7%). LR models suggested apparent protective associations for having 1 to 2 abnormal indicators (adjusted odds ratios, 0.58-0.79), although formal interaction tests were not statistically significant (all *P* > 0.05) (**Table 6**). This counterintuitive clinical phenomenon is not attributable to the direct physiological effects of the biomarkers, but rather arises from real-world monitoring bias. Specifically, patients with abnormal biomarkers may receive more intensive monitoring and conservative insulin titration, thereby reducing the risk of hypoglycemia (**Figure 9**).

**Table 5:** Priority risk triage categories based on biomarker abnormality flags.

Priority group	*n*	Event rate	Recommendation
1: Normal	194	0.557	Routine monitoring
2: Moderate	22	0.545	Increase monitoring and review abnormal labs
3: High	194	0.515	High-risk monitoring and targeted correction
4: Critical	40	0.425	Urgent clinician review and corrective actions

**Table 6:** Joint biomarker effects.

*n*_abnormal	*n*	Events	Event rate (%)	OR	*CI* Lower	*CI* Upper	*P* value
0	194	108	55.7	/	/	/	/
1	216	112	51.9	0.789	0.502	1.239	0.303
2	40	17	42.5	0.584	0.268	1.272	0.175

Hypoglycemia Frequency Stratified by the Number of Abnormal Hemoglobin, Potassium, and Sodium Indicators.

### 3.7 Comparison of clinical utility between random forest model and biomarker threshold-based rule

**Figure 10:**
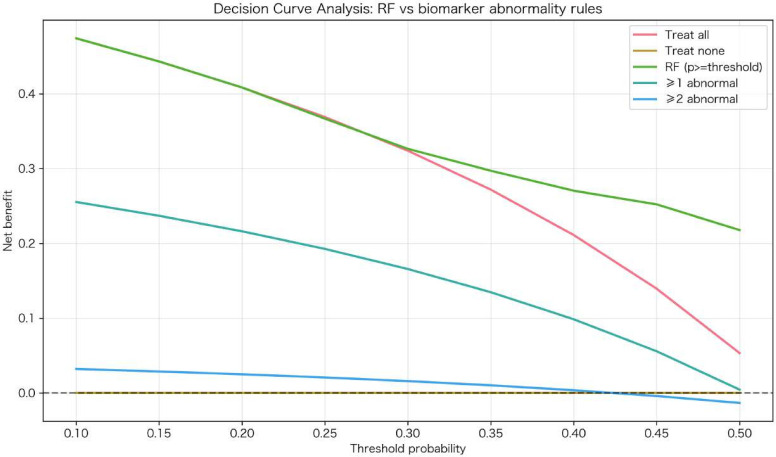
Decision curve analysis comparing the random forest model with biomarker abnormality-based rules. Clinical usefulness (net benefit) was compared across threshold probabilities of 0.10–0.50 for the treat all/treat none baselines, the RF probability rule (treat when predicted risk ≥ threshold), and the rule-based strategies (treat when ≥1 biomarker abnormal; treat when ≥2 biomarkers abnormal), with higher net benefit at a given threshold indicating a superior strategy for the corresponding clinical tradeoff.

Decision curve analysis comparing RF probability-based strategies to simple biomarker rules showed that rule-based approaches (treating patients with ≥1 or ≥2 abnormal biomarkers) provided consistently lower net benefit across most clinically relevant thresholds. At a 10% risk threshold, the net benefit was 0.474 for the RF model vs 0.255 for the ≥1 abnormal biomarker rule. Across thresholds from 0.10 to 0.50, the RF model outperformed biomarker rules and “treat all/none” strategies, indicating that continuous risk prediction offers a more favorable benefit-harm balance than dichotomous biomarker cutoffs (**Figure 10**).

## 4 Discussion

### 4.1 Performance advantages and innovative value of a population-customized interpretable model for in-hospital hypoglycemia prediction

Based on multicenter data, this study developed and validated an interpretable machine learning prediction model for in-hospital hypoglycemia in Chinese inpatients with T1DM. Among the four evaluated algorithms, the RF model exhibited the optimal performance by virtue of the inherent characteristics of ensemble learning algorithms, including its robust processing of multidimensional clinical and laboratory data, effective capture of complex nonlinear variable correlations and interaction effects, as well as avoidance of overfitting through bootstrapping and random feature selection. Following systematic hyperparameter optimization, the model achieved a good consistency between predicted probabilities and observed outcomes across all risk quantile groups. Furthermore, this study identified the relative importance and directional effects of key predictive factors via SHAP analysis, and combined clinical risk stratification based on predicted probability thresholds to provide a quantitative basis for targeted hypoglycemia interventions. Ultimately, the model achieved a favorable balance between predictive accuracy and clinical practicability. It can not only serve as a decision-support tool for personalized hypoglycemia management in hospitalized T1DM patients, but also offer practical references for the application of ensemble learning algorithms in addressing complex clinical issues and the development of population-specific prediction models.

This study was designed to address the unsolved key issues in previous research on hypoglycemia prediction in T1DM, and its innovative value is reflected in the targeted breakthroughs in these problems. Aiming at the insufficient population adaptability of previous studies, this study abandoned the research design dominated by European Americans (11, 21) or community-dwelling T1DM patients (22, 23) and focused on the population of Chinese hospitalized T1DM patients. Considering the ethnic heterogeneities in genetic background, dietary habits and treatment strategies between Chinese and Western populations (24), as well as the specific risk characteristics of Chinese hospitalized T1DM patients including reduced lean body mass, impaired glycogen storage capacity and increased sensitivity to insulin-induced hypoglycemia (25, 26), the customized model developed in this study effectively makes up for the lack of generalizability of Western-derived models in Chinese hospitalized patients.

To overcome the limitations in study design and data of previous research, this study adopted a multicenter design to enroll 1,498 participants from five tertiary hospitals and integrated 65 candidate predictive factors covering comprehensive clinical and laboratory indicators, replacing the previous research mode characterized by single-center small sample size and sole reliance on outpatient follow-up or self-monitored blood glucose data (27–29). This design effectively avoided sample homogenization, significantly improved the external validity and generalization ability of the model, and was more in line with the clinical characteristics of in-hospital hypoglycemia risk in inpatients, which is synergistically driven by comorbidities, aggressive insulin therapy and acute clinical events (30, 31).

Targeting the methodological limitations of traditional linear models, this study employed a RF model in lieu of conventional linear approaches. The RF model enables efficient processing of high-dimensional data related to in-hospital hypoglycemia risk, including glycemic variability metrics and latest available laboratory values preceding hypoglycemic episodes, while effectively capturing complex nonlinear interactions among predictive variables (22, 32). Meanwhile, SHAP analysis was innovatively integrated to quantify the contribution of each predictive factor, clarify its directional effect and interaction mechanism, thereby solving the problem of insufficient interpretability caused by the “black box” characteristic of previous machine learning models (21, 33). This allows clinicians to clearly understand the basis for prediction and directly translate the model output into clinical decisions (34). To further improve the model and alleviate concerns about the “black box”, we derived an exploratory points-based triage score from a parsimonious logistic regression model using the main SHAP-identified predictors. As a practical bedside aid, the specific content of this points-based triage score is presented in **Supplementary Table 3**, facilitating its quick reference and application by clinical personnel. It should be clearly stated that this points-based score only serves as a simplified auxiliary tool for manual risk estimation, while the probability output of the random forest model remains the primary model for full electronic implementation. The two have clear positioning and play complementary roles.

In this study, the RF model achieved a favorable AUC value in the external validation cohort. This performance advantage stems not merely from the algorithm selection, but more from the high consistency of study design, model construction with the complex clinical environment of hospitalized patients. Integrating real-time clinical indicators, the model accurately captures acute fluctuations in in-hospital hypoglycemia risk, advancing from universal prediction to hospital-specific, scenario-specific and population-specific prediction (35).

### 4.2 Implications of risk stratification and decision curve analysis

The risk stratification results of the optimal RF model provide a reliable basis for the stratified management of hospitalized patients with T1DM. By defining clear risk groups, clinicians can allocate medical resources in a targeted manner, implement intensive interventions for high-risk patients, and avoid over-intervention in low-risk groups, thereby optimizing the efficiency of resource utilization. DCA demonstrates this model’s clinical utility, outperforming traditional empirical approaches, overcoming uniform intervention drawbacks, and aligning with precision medicine core concepts. Compared with previous hypoglycemia models based on LR, this model maintains superior performance over a wider range of risk thresholds by virtue of the algorithm’s ability to capture non-linear relationships and integrate multi-dimensional predictive indicators (36).

Moreover, it is specifically tailored to the clinical characteristics of hospitalized T1DM patients, further enhancing its relevance and reliability in practical application. Currently, clinical hypoglycemia prevention mainly relies on empirical judgment. Although continuous glucose monitoring (CGM) technology has obvious advantages in early warning of hypoglycemia, its widespread application is hindered by popularization bottlenecks, resulting in insufficient accuracy in clinical interventions (37). This model achieves stratified intervention through quantitative risk assessment, which is highly compatible with the current status of clinical practice in China. It can not only make up for the shortcomings of existing intervention models but also improve patient prognosis by optimizing resource allocation and reducing the incidence of hypoglycemia, demonstrating significant clinical transformation value.

### 4.3 Feature analysis extension and clinical mechanistic interpretation based on SHAP values

SHAP analysis of the optimal random forest model identified the relative importance of core features associated with in-hospital hypoglycemia among Chinese patients with T1DM. As a *post hoc* model interpretability approach, SHAP facilitated the visualization of nonlinear relationships and feature interactions within the trained model, rather than inferring causal relationships. To enhance clinical applicability, we further translated the top-ranked SHAP features into actionable clinical high-risk personas. These translational findings improved model transparency and real-world interpretability, with all feature-hypoglycemia associations supported by existing clinical and physiological plausibility.

Hemoglobin is a key indicator of nutritional status and chronic disease severity (38), and was ranked as the top predictive feature by SHAP analysis, corresponding to the malnourished/low hemoglobin high-risk profile. It has been biologically plausibly linked to hypoglycemia through two potential pathways. Low hemoglobin may reflect malnutrition, which could downregulate hepatic glycogen synthase and attenuate counterregulatory responses such as glucagon and epinephrine, thereby potentially impairing glucose counter regulation. Abnormally elevated hemoglobin may increase blood viscosity and impair perfusion in key glucose-regulating organs such as the liver and pancreas, potentially contributing to disrupted systemic glucose homeostasis (39, 40).

Electrolytes including potassium and sodium, which are the second and third most important SHAP features, represent the electrolyte imbalance high-risk profile, which is highly correlated with an increased risk of hypoglycemia. These electrolytes are involved in glucose metabolism through complex regulatory pathways and show important interactive effects (41). Potassium modulates pancreatic β-cell insulin secretion via calcium influx and mediates GLUT4-dependent glucose transport (42), both hypokalemia and hyperkalemia may disrupt these processes and potentially increase hypoglycemia risk. Sodium maintains osmotic balance and cellular integrity, and disturbances in sodium metabolism may impair glycogen synthesis, insulin release, and insulin receptor signaling (43).

LDL-C may influence hypoglycemia risk through vascular and cellular mechanisms. Elevated LDL-C has been associated with pancreatic microvascular endothelial dysfunction and β-cell oxidative stress, which could reduce insulin secretory reserve (44). Low LDL-C levels may impair steroid hormone synthesis and hepatic glycogen storage. Both abnormalities may confer an increased risk of hypoglycemia (45).

Age at onset may influence hypoglycemia risk through long-term effects on glucose regulatory systems. Patients with onset age ≤ 18 years often have a long disease duration and may experience progressive β-cell failure (46). Those with onset age ≥ 40 years frequently have comorbidities including hypertension and renal insufficiency, which may alter insulin clearance and organ perfusion, as well as lower skeletal muscle mass that reduces glycogen storage capacity. These factors may collectively contribute to increased hypoglycemia risk (47).

SHAP dependence plots showed a significant U-shaped nonlinear relationship between key features and hypoglycemia risk. Hemoglobin, potassium and sodium followed this pattern, with the lowest risk within the normal range and a sharp rise when deviated from it, indicating the need to maintain these indices in the normal range rather than only correcting extreme abnormalities. Guided by the aforementioned clinical high-risk personas, most core clinical predictive features are modifiable. Corresponding management recommendations include routine hemoglobin monitoring, targeted nutritional support for anemia and appropriate fluid replacement for high levels, daily routine electrolytes monitoring with timely and accurate correction of abnormalities, standard statin use for elevated low-density lipoprotein cholesterol and individualized nutritional intervention for excessively low levels, as well as precision individualized insulin dosage adjustment for early-onset patients and systematic comprehensive comorbidities management for late-onset ones. Comprehensive management of interactive features effectively avoids synergistic risk amplification and serves as a key measure for reducing in-hospital hypoglycemia incidence.

In summary, the core predictive features identified by SHAP analysis are tightly associated with the pathophysiological basis of in-hospital hypoglycemia in T1DM patients. By translating these features into clinically intuitive high-risk personas, we clarified their importance hierarchy, U-shaped correlations and interactive effects, yielding actionable bedside clinical guidance. Strengthening dynamic monitoring and integrated management of these modifiable features to maintain normality is expected to effectively reduce in-hospital hypoglycemia incidence and enhance inpatient safety.

### 4.4 Monitoring and intervention strategies for hypoglycemia risk in T1DM based on biomarkers

This study combined statistical analysis with machine learning models to optimize hypoglycemia risk monitoring and intervention strategies for hospitalized T1DM patients, and formulated corresponding clinical priority rules.

A counterintuitive association was the core finding. Patients with normal biomarkers had the highest hypoglycemia risk, while those with 1–2 abnormal indicators showed a statistical protective effect. This was not due to a genuine protective role of abnormal indicators but surveillance bias. Patients with abnormal biomarkers received prioritized clinical attention, including more frequent testing and conservative insulin dose adjustments, which objectively reduced hypoglycemia risk (48).

Essentially, such abnormalities reflected the body’s physiological compensatory mechanisms rather than primary hypoglycemia risk drivers. *Post-hoc* analysis was performed to define optimal indicator reference ranges by identifying risk nadirs on RCS curves. This verified the aforementioned association and redefined clinical focus. Clinical intervention should shift from treating abnormal indicators to strengthening surveillance for high-risk patients with normal biomarkers.

A stratified intervention protocol was optimized based on these findings. Priority 1 emergent intervention applies to patients with 3 abnormal indicators or 2 including serum potassium. This group requires prioritized potassium metabolism correction, concurrent anemia screening and increased blood glucose monitoring frequency. Priority 2 routine intervention targets those with 1–2 abnormal indicators, focusing on electrolyte disorder correction with subsequent anemia management if hemoglobin < 100 g/L. Priority 3 conservative intervention is for patients with biomarkers in the yellow zone, involving conservative treatment and enhanced hypoglycemia monitoring.

This strategy translates model-derived findings into standardized clinical workflows. It effectively reduces oversight of high-risk populations from surveillance bias and provides a basis for precise in-hospital T1DM hypoglycemia prevention and control.

### 4.5 Model robustness and generalizability verification

External validation results fully confirmed the robust performance of the optimal RF model. In the external validation cohort, the model maintained stable discriminative ability. Furthermore, it exhibited strong robustness even under class imbalance, outperforming the baseline significantly on the Precision-Recall Curve. This stable cross-institutional performance demonstrates the model’s favorable cross-institutional adaptability, laying a solid foundation for its wide clinical application.

Notably, the balanced baseline characteristics between the development and the external validation cohorts further guarantees the model’s generalizability. The consistent distribution of potential confounding factors in the two cohorts avoids selection bias and ensures that the model’s predictive rules can be reliably extrapolated to other similar inpatient populations with T1DM.

### 4.6 Limitations and future perspectives

This study has inherent limitations. First, its retrospective design introduces unavoidable selection bias, impairing model generalizability. Second, inadequate collection of core clinical information includes excluded incomplete CGM data and insufficient records of comorbidities, lifestyles and insulin regimens. This leads to incomplete predictive features, potentially omitting key factors and compromising result reliability and applicability. Third, although we performed external validation within the participating hospitals, additional validation in other health systems and ethnic populations is required before broad implementation.

Future work should include prospective multicenter studies to evaluate the impact of RF-based risk prediction on clinical outcomes, workflow, and costs; integration of richer multimodal data including continuous glucose monitoring, medication administration records, and nursing documentation; and development of user-friendly decision support tools embedded in hospital information systems. Such tools could deliver real-time risk estimates at the bedside and support personalized intervention strategies for inpatients with T1DM.

## 5 Conclusions

In this multicenter retrospective study of Chinese adults with T1DM, we developed and externally validated an interpretable RF model for predicting in-hospital hypoglycemia using routine electronic health record data. The model showed strong discrimination, good calibration, useful risk stratification and net clinical benefit across decision thresholds, and may help clinicians identify high-risk patients, allocate surveillance resources efficiently and implement targeted preventive interventions.

## Data Availability

The raw data supporting the conclusions of this article will be made available by the authors, without undue reservation.
