# Supplementary figures and images for: Development and validation of an interpretable machine learning model for predicting in-hospital hypoglycemia in adults with type 1 diabetes mellitus: a multicenter retrospective study
Source: Front Endocrinol (Lausanne). 2026 Apr 17;17:1816599. doi: 10.3389/fendo.2026.1816599 (PMC13140310; doi:10.3389/fendo.2026.1816599)

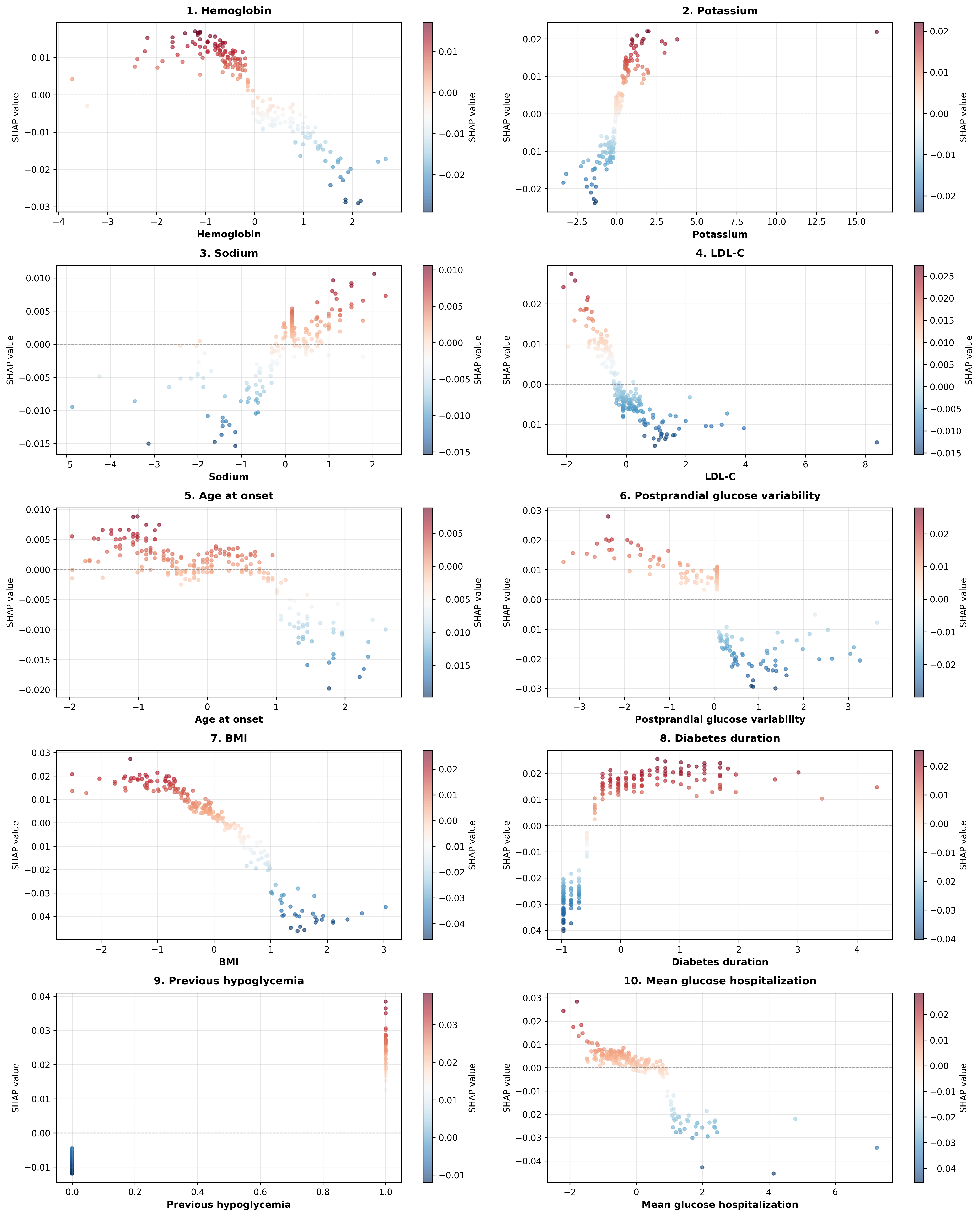

Supplement: Supplementary file 1 [file Image1.tiff]
