# Supplementary material for: Development and validation of an interpretable machine learning model for predicting in-hospital hypoglycemia in adults with type 1 diabetes mellitus: a multicenter retrospective study
Source: Front Endocrinol (Lausanne). 2026 Apr 17;17:1816599. doi: 10.3389/fendo.2026.1816599 (PMC13140310; doi:10.3389/fendo.2026.1816599)
Supplement: Supplementary file 2 [file Table1.docx]

**Supplementary Table 1 List of 65 features and associated metadata**

| **Feature** | **Encoding** | **Data Type** | **Category** | **Minimum** | **Maximum** | **Cardinality** | **Missing (%)** | **Included Post Missingness Filter** |
| --- | --- | --- | --- | --- | --- | --- | --- | --- |
| BMI | Numerical | float64 | Continuous/Ordinal | 11.96 | 52.6 | 548 | 3.629 | Yes |
| Hospital_Days | Numerical | int64 | Continuous/Ordinal | 1 | 52 | 34 | 0 | Yes |
| Onset_Age | Numerical | int64 | Continuous/Ordinal | 2 | 84 | 75 | 0 | Yes |
| Disease_Duration | Numerical | float64 | Continuous/Ordinal | / | / | 38 | 0 | Yes |
| Premeal_Insulin_Dose | Numerical | float64 | Continuous/Ordinal | 0 | 76 | 62 | 2.961 | Yes |
| Ppge_Glucose_Variability | Numerical | float64 | Continuous/Ordinal | / | / | 335 | 37.631 | Yes |
| Lage_Glucose_Variability | Numerical | float64 | Continuous/Ordinal | / | / | 210 | 0.955 | Yes |
| Mean_Glucose_Hospital | Numerical | float64 | Continuous/Ordinal | 4.26 | 28.1 | 555 | 0 | Yes |
| Serum_Creatinine | Numerical | float64 | Continuous/Ordinal | 15.7 | 1550.1 | 478 | 1.624 | Yes |
| Total_Protein | Numerical | float64 | Continuous/Ordinal | 4.72 | 99 | 325 | 3.534 | Yes |
| Triglycerides | Numerical | float64 | Continuous/Ordinal | / | / | 279 | 5.731 | Yes |
| LDL_Cholesterol | Numerical | float64 | Continuous/Ordinal | 0.32 | 10.65 | 340 | 5.826 | Yes |
| HDL_Cholesterol | Numerical | float64 | Continuous/Ordinal | 0.28 | 96 | 180 | 5.922 | Yes |
| Hemoglobin | Numerical | float64 | Continuous/Ordinal | 4.84 | 192 | 105 | 0.955 | Yes |
| RBC_Count | Numerical | float64 | Continuous/Ordinal | 1.98 | 160 | 284 | 0.955 | Yes |
| Vitamin_D | Numerical | float64 | Continuous/Ordinal | / | / | 362 | 55.396 | No (>40% missing) |
| Potassium | Numerical | float64 | Continuous/Ordinal | 1.87 | 11.6 | 207 | 21.012 | Yes |
| Sodium | Numerical | float64 | Continuous/Ordinal | 115.8 | 149.1 | 160 | 21.012 | Yes |
| Calcium | Numerical | float64 | Continuous/Ordinal | 1.05 | 3.09 | 97 | 21.012 | Yes |
| Gender | Categorical | object | Nominal/Ordinal | / | / | 2 | 0 | Yes |
| Age | Categorical | object | Nominal/Ordinal | / | / | 68 | 0 | Yes |
| Marital_Status | Categorical | object | Nominal/Ordinal | / | / | 4 | 0 | Yes |
| Ethnicity | Categorical | object | Nominal/Ordinal | / | / | 19 | 0 | Yes |
| Education | Categorical | object | Nominal/Ordinal | / | / | 5 | 0 | Yes |
| Insurance_Type | Categorical | object | Nominal/Ordinal | / | / | 3 | 0 | Yes |
| Smoking_History | Categorical | object | Nominal/Ordinal | / | / | 2 | 0 | Yes |
| Smoking_Years | Categorical | object | Nominal/Ordinal | / | / | 39 | 58.453 | No (>40% missing) |
| Alcohol_History | Categorical | object | Nominal/Ordinal | / | / | 2 | 0 | Yes |
| Alcohol_Years | Categorical | object | Nominal/Ordinal | / | / | 28 | 77.077 | No (>40% missing) |
| T1DM_Subtype | Categorical | object | Nominal/Ordinal | / | / | 3 | 0 | Yes |
| Previous_Hypoglycemia | Categorical | object | Nominal/Ordinal | / | / | 2 | 4.298 | Yes |
| DM_Family_History | Categorical | object | Nominal/Ordinal | / | / | 2 | 0 | Yes |
| Hypertension_History | Categorical | object | Nominal/Ordinal | / | / | 2 | 0 | Yes |
| Diabetic_Ketosis | Categorical | object | Nominal/Ordinal | / | / | 2 | 0 | Yes |
| Diabetic_Ketoacidosis | Categorical | object | Nominal/Ordinal | / | / | 2 | 27.316 | Yes |
| Diabetic_Retinopathy | Categorical | object | Nominal/Ordinal | / | / | 2 | 0 | Yes |
| Diabetic_Nephropathy | Categorical | object | Nominal/Ordinal | / | / | 2 | 0 | Yes |
| Diabetic_Neuropathy | Categorical | object | Nominal/Ordinal | / | / | 2 | 0 | Yes |
| Diabetic_Vasculopathy | Categorical | object | Nominal/Ordinal | / | / | 2 | 0 | Yes |
| Diabetic_Foot | Categorical | object | Nominal/Ordinal | / | / | 2 | 0 | Yes |
| Total_Complications | Categorical | int64 | Nominal/Ordinal | / | / | 9 | 0 | Yes |
| Sleep_Disorder | Categorical | object | Nominal/Ordinal | / | / | 2 | 0 | Yes |
| Polypharmacy | Categorical | object | Nominal/Ordinal | / | / | 2 | 0 | Yes |
| Oral_Antidiabetic | Categorical | object | Nominal/Ordinal | / | / | 2 | 0 | Yes |
| Insulin_Regimen | Categorical | object | Nominal/Ordinal | / | / | 8 | 2.483 | Yes |
| Insulin_Pump_Use | Categorical | object | Nominal/Ordinal | / | / | 2 | 0 | Yes |
| Total_Insulin_Dose | Categorical | object | Nominal/Ordinal | / | / | 153 | 2.865 | Yes |
| Longacting_Insulin_Dose | Categorical | object | Nominal/Ordinal | / | / | 91 | 2.961 | Yes |
| Insulin_Types | Categorical | object | Nominal/Ordinal | / | / | 3 | 2.292 | Yes |
| HbA1c | Categorical | object | Nominal/Ordinal | / | / | 194 | 3.534 | Yes |
| Fasting_Cpeptide | Categorical | object | Nominal/Ordinal | / | / | 209 | 18.052 | Yes |
| Cpeptide_30min | Categorical | object | Nominal/Ordinal | / | / | 195 | 55.205 | No (>40% missing) |
| Cpeptide_1h | Categorical | object | Nominal/Ordinal | / | / | 218 | 52.053 | No (>40% missing) |
| Cpeptide_2h | Categorical | object | Nominal/Ordinal | / | / | 273 | 38.873 | Yes |
| EGFR | Categorical | object | Nominal/Ordinal | / | / | 891 | 1.624 | Yes |
| Total_Cholesterol | Categorical | object | Nominal/Ordinal | / | / | 428 | 5.635 | Yes |
| WBC_Count | Categorical | object | Nominal/Ordinal | / | / | 603 | 0.955 | Yes |
| CRP | Categorical | object | Nominal/Ordinal | / | / | 267 | 41.547 | No (>40% missing) |
| Urine_Microalbumin | Categorical | object | Nominal/Ordinal | / | / | 347 | 44.222 | No (>40% missing) |
| Urine_Protein | Categorical | object | Nominal/Ordinal | / | / | 6 | 17.574 | Yes |
| GAD_Antibody | Categorical | object | Nominal/Ordinal | / | / | 2 | 49.761 | No (>40% missing) |
| ZNT8_Antibody | Categorical | object | Nominal/Ordinal | / | / | 2 | 80.325 | No (>40% missing) |
| IAA_Antibody | Categorical | object | Nominal/Ordinal | / | / | 2 | 49.666 | No (>40% missing) |
| IA2_Antibody | Categorical | object | Nominal/Ordinal | / | / | 2 | 80.325 | No (>40% missing) |
| ICA_Antibody | Categorical | object | Nominal/Ordinal | / | / | 2 | 81.948 | No (>40% missing) |
