# Supplementary material for: Development and validation of an interpretable machine learning model for predicting in-hospital hypoglycemia in adults with type 1 diabetes mellitus: a multicenter retrospective study
Source: Front Endocrinol (Lausanne). 2026 Apr 17;17:1816599. doi: 10.3389/fendo.2026.1816599 (PMC13140310; doi:10.3389/fendo.2026.1816599)
Supplement: Supplementary file 3 [file Table2.docx]

Supplementary Table 2 Model decision thresholds

| **Model** | **Decision Threshold** | **Validation AUC** | **Validation Accuracy** | **Validation Sensitivity** | **Validation Specificity** | **Validation F1 Score** | **Validation Brier Score** |
| --- | --- | --- | --- | --- | --- | --- | --- |
| Logistic Regression | 0.615 | 0.817 | 0.738 | 0.622 | 0.869 | 0.715 | 0.177 |
| Decision Tree | 0.644 | 0.749 | 0.705 | 0.658 | 0.758 | 0.702 | 0.214 |
| Random Forest | 0.556 | 0.820 | 0.757 | 0.640 | 0.889 | 0.736 | 0.191 |
| XGBoost | 0.430 | 0.796 | 0.733 | 0.865 | 0.586 | 0.774 | 0.185 |

Note: Decision thresholds were determined using the Youden index during internal validation
