# Supplementary material for: Development and validation of an interpretable machine learning model for predicting in-hospital hypoglycemia in adults with type 1 diabetes mellitus: a multicenter retrospective study
Source: Front Endocrinol (Lausanne). 2026 Apr 17;17:1816599. doi: 10.3389/fendo.2026.1816599 (PMC13140310; doi:10.3389/fendo.2026.1816599)
Supplement: Supplementary file 4 [file Table3.docx]

Supplementary Table 3a Exploratory bedside points score

| **Component** | **Rule** | **Points** | **Rationale** |
| --- | --- | --- | --- |
| Hemoglobin | < 120 or > 160 g/L | 2 | Top SHAP predictor with U-shaped risk |
| Potassium | < 3.5 or > 5.0 mmol/L | 2 | Top SHAP predictor with U-shaped risk |
| Sodium | < 135 or > 145 mmol/L | 2 | Top SHAP predictor with U-shaped risk |
| BMI | < 18.5 or > 24.0 kg/m^2^ | 1 | Nonlinear association in SHAP/RCS |
| Previous hypoglycemia | Yes | 2 | High-importance history feature |
| LAGE glucose variability | > 6 mmol/L | 1 | High SHAP contribution |

Supplementary Table 3b Score risk bands

| **Total Points Range** | **Triage Level** | **Recommended Clinical Action** |
| --- | --- | --- |
| 0-2 | Lower risk | Routine glucose surveillance |
| 3-5 | Intermediate risk | Increase glucose checks and medication review |
| 6-10 | Higher risk | Intensive monitoring and proactive prevention |
