# Supplementary material for: Development and validation of an interpretable machine learning model for predicting in-hospital hypoglycemia in adults with type 1 diabetes mellitus: a multicenter retrospective study
Source: Front Endocrinol (Lausanne). 2026 Apr 17;17:1816599. doi: 10.3389/fendo.2026.1816599 (PMC13140310; doi:10.3389/fendo.2026.1816599)
Supplement: Supplementary file 5 [file Table4.docx]

**Per-fold performance metrics (all folds × all models)**

| **Fold** | **Model** | **AUC** | **Accuracy** | **Sensitivity** | **Specificity** | **Precision** | **F1** | **Brier** |
| --- | --- | --- | --- | --- | --- | --- | --- | --- |
| 1 | DT | 0.599 | 0.605 | 0.745 | 0.450 | 0.599 | 0.664 | 0.242 |
| 2 | DT | 0.591 | 0.543 | 0.721 | 0.343 | 0.552 | 0.625 | 0.244 |
| 3 | DT | 0.622 | 0.603 | 0.918 | 0.253 | 0.577 | 0.709 | 0.235 |
| 4 | DT | 0.500 | 0.526 | 1.000 | 0.000 | 0.526 | 0.690 | 0.249 |
| 5 | DT | 0.583 | 0.555 | 0.555 | 0.556 | 0.581 | 0.567 | 0.256 |
| 1 | LR | 0.827 | 0.767 | 0.791 | 0.740 | 0.770 | 0.780 | 0.169 |
| 2 | LR | 0.808 | 0.733 | 0.730 | 0.737 | 0.757 | 0.743 | 0.181 |
| 3 | LR | 0.828 | 0.794 | 0.827 | 0.758 | 0.791 | 0.809 | 0.170 |
| 4 | LR | 0.801 | 0.732 | 0.791 | 0.667 | 0.725 | 0.757 | 0.188 |
| 5 | LR | 0.776 | 0.699 | 0.709 | 0.687 | 0.716 | 0.712 | 0.206 |
| 1 | RF | 0.778 | 0.724 | 0.773 | 0.67 | 0.720 | 0.746 | 0.220 |
| 2 | RF | 0.847 | 0.748 | 0.874 | 0.606 | 0.713 | 0.785 | 0.213 |
| 3 | RF | 0.846 | 0.737 | 0.791 | 0.677 | 0.731 | 0.760 | 0.213 |
| 4 | RF | 0.790 | 0.694 | 0.845 | 0.525 | 0.664 | 0.744 | 0.219 |
| 5 | RF | 0.768 | 0.694 | 0.782 | 0.596 | 0.683 | 0.729 | 0.219 |
| 1 | XGBoost | 0.820 | 0.724 | 0.773 | 0.67 | 0.720 | 0.746 | 0.181 |
| 2 | XGBoost | 0.818 | 0.710 | 0.757 | 0.657 | 0.712 | 0.734 | 0.179 |
| 3 | XGBoost | 0.828 | 0.766 | 0.791 | 0.737 | 0.770 | 0.780 | 0.170 |
| 4 | XGBoost | 0.818 | 0.742 | 0.836 | 0.636 | 0.719 | 0.773 | 0.182 |
| 5 | XGBoost | 0.806 | 0.718 | 0.718 | 0.717 | 0.738 | 0.728 | 0.187 |

**Summary across folds (Mean ± SD; 95% CI)**

| **Model** | **AUC** | **Accuracy** | **Sensitivity** | **Specificity** | **Precision** | **F1** | **Brier (lower better)** |
| --- | --- | --- | --- | --- | --- | --- | --- |
| LR | 0.808 ± 0.021 [0.781, 0.835] | 0.745 ± 0.037 [0.700, 0.790] | 0.770 ± 0.049 [0.709, 0.830] | 0.718 ± 0.039 [0.669, 0.766] | 0.752 ± 0.031 [0.713, 0.791] | 0.760 ± 0.037 [0.715, 0.806] | 0.183 ± 0.015 [0.164, 0.202] |
| RF | 0.806 ± 0.038 [0.758, 0.853] | 0.719 ± 0.025 [0.689, 0.750] | 0.813 ± 0.044 [0.758, 0.868] | 0.615 ± 0.062 [0.538, 0.692] | 0.702 ± 0.028 [0.668, 0.737] | 0.753 ± 0.021 [0.726, 0.779] | 0.217 ± 0.003 [0.212, 0.221] |
| XGBoost | 0.818 ± 0.008 [0.808, 0.828] | 0.732 ± 0.022 [0.704, 0.759] | 0.775 ± 0.044 [0.721, 0.829] | 0.683 ± 0.042 [0.631, 0.736] | 0.732 ± 0.023 [0.703, 0.761] | 0.752 ± 0.023 [0.723, 0.781] | 0.180 ± 0.006 [0.172, 0.187] |
| DT | 0.579 ± 0.047 [0.521, 0.637] | 0.566 ± 0.036 [0.522, 0.611] | 0.788 ± 0.175 [0.570, 1.0000] | 0.320 ± 0.212 [0.057, 0.584] | 0.567 ± 0.028 [0.532, 0.602] | 0.651 ± 0.056 [0.581, 0.721] | 0.245 ± 0.008 [0.236, 0.255] |

**Note:** All metrics shown as Mean ± Standard Deviation (95% Confidence Interval)

**Fold distribution checks (Stratification and shift indicators)**

| **Fold** | **Train N** | **Val N** | **Train Pos Rate** | **Val Pos Rate** | **Train Avg Missing** | **Val Avg Missing** | **Processed Features** | **Max SMD**  **(top 10 numeric)** | **Max TVD (sample 5 categorical)** |
| --- | --- | --- | --- | --- | --- | --- | --- | --- | --- |
| 1 | 837 | 210 | 0.527 | 0.524 | 0.064 | 0.059 | 3107 | 0.162 | 0.256 |
| 2 | 837 | 210 | 0.526 | 0.529 | 0.065 | 0.057 | 3119 | 0.116 | 0.268 |
| 3 | 838 | 209 | 0.526 | 0.526 | 0.078 | 0.081 | 3128 | 0.132 | 0.245 |
| 4 | 838 | 209 | 0.526 | 0.526 | 0.070 | 0.075 | 3139 | 0.149 | 0.262 |
| 5 | 838 | 209 | 0.526 | 0.526 | 0.077 | 0.083 | 3179 | 0.122 | 0.228 |
