# Supplementary material for: Development and validation of an interpretable machine learning model for predicting in-hospital hypoglycemia in adults with type 1 diabetes mellitus: a multicenter retrospective study
Source: Front Endocrinol (Lausanne). 2026 Apr 17;17:1816599. doi: 10.3389/fendo.2026.1816599 (PMC13140310; doi:10.3389/fendo.2026.1816599)
Supplement: Supplementary file 6 [file Table5.docx]

**Definition of biomarker thresholds based on clinical and spline plot findings**

In this study, exploratory normal thresholds were established based on clinical judgment and findings from U-shaped restricted cubic spline plots, which were 110-140 g/L for hemoglobin, 3.5-4.4 mmol/L for serum potassium, and 135-140 mmol/L for serum sodium, respectively.

Metrics computed from RF predicted probabilities on the external validation cohort

| **AUC** | **Accuracy** | **Sensitivity** | **Specificity** | **Precision** | **F1** | **Brier** | **N** | **Positives** |
| --- | --- | --- | --- | --- | --- | --- | --- | --- |
| 0.766 | 0.691 | 0.831 | 0.535 | 0.666 | 0.739 | 0.211 | 450 | 237 |

*The area under the curve (AUC) was approximately 0.766, indicating a moderate-to-good discriminative ability of the model in external validation. With sensitivity (0.831) higher than specificity (0.535), the model showed a stronger tendency to identify positive samples at the default cutoff. Specifically, it captured a larger proportion of positive cases, but also misclassified a certain number of negative samples as positive ones. The Brier score was around 0.211, which reflects the calibration degree and overall probability prediction accuracy of the model. A smaller Brier score indicates better model performance, and its specific interpretation should be combined with the characteristics of the dataset. These metrics provide evidence for biomarker selection: some biomarkers may have a statistical correlation with the outcome, but their practical predictive value is limited. The predictive model constructed in this study exhibits distinct application efficacy, which can enhance the reference significance of biomarker effect analysis for risk stratification and clinical threshold determination.*

Adjusted hypoglycemia odds ratios at selected hemoglobin thresholds

| **Threshold** | **Value** | **OR (95% *CI*)** | ***P* value** |
| --- | --- | --- | --- |
| value_90 | 90.0 | 1.330 (0.707–2.501) | 0.376 |
| value_100 | 100.0 | 1.255 (0.760–2.073) | 0.375 |
| value_110 | 110.0 | 1.185 (0.817–1.718) | 0.371 |
| value_120 | 120.0 | 1.118 (0.878–1.424) | 0.365 |

**Hemoglobin (reference = 139.0)**

*This table presents adjusted odds ratios (ORs) for hypoglycemia at specific hemoglobin (Hb) values (90, 100, 110, 120), with 139.0 as the reference value (consistent with the biomarker summary table). Derived from the fitted restricted cubic spline (RCS) logistic model, these estimates offer clinical interpretability. Lower Hb values show slightly elevated ORs (>1) relative to the reference, but all P values (0.365–0.376) are large and confidence intervals (CIs) are wide and cross 1, indicating decreasing Hb correlates with a trend toward higher OR yet with substantial uncertainty. Aligned with Hb’s non-significant P(overall), these results confirm no robust statistical evidence for risk changes at the tested Hb thresholds after covariate adjustment, supporting a lower priority for Hb in threshold-based clinical decision rules in this dataset.*

Adjusted hypoglycemia odds ratios at selected potassium thresholds

| **Threshold** | **Value** | **OR (95% *CI*)** | ***P* value** |
| --- | --- | --- | --- |
| value_3 | 3.0 | 0.625 (0.309–1.264) | 0.191 |
| value_3.5 | 3.5 | 0.781 (0.541–1.130) | 0.190 |
| value_4 | 4.0 | 0.978 (0.946–1.010) | 0.174 |
| value_4.5 | 4.5 | 1.288 (0.995–1.666) | 0.0543 |
| value_5 | 5.0 | 1.796 (0.787–4.096) | 0.164 |

**Potassium (reference = 4.05)**

*Adjusted hypoglycemia odds ratios (ORs) for potassium values 3.0–5.0 (reference 4.05, consistent with biomarker summary). With P (overall)=0.173, low potassium (3.0, 3.5) shows OR < 1 and high potassium (4.5, 5.0) shows OR > 1; 4.5 has a borderline P-value (~0.0543), and 5.0 has wide CIs (0.787–4.096). Potassium’s graded OR pattern makes it a better follow-up candidate than hemoglobin, despite non-significant results across all thresholds.*

Adjusted hypoglycemia odds ratios at selected sodium thresholds

| **Threshold** | **Value** | **OR (95% *CI*)** | ***P* value** |
| --- | --- | --- | --- |
| value_130 | 130.0 | 0.541 (0.324–0.903) | 0.0188 |
| value_135 | 135.0 | 0.768 (0.616–0.956) | 0.0182 |
| value_140 | 140.0 | 1.062 (0.999–1.129) | 0.0525 |
| value_145 | 145.0 | 1.162 (0.585–2.307) | 0.669 |

**Sodium (reference = 138.8)**

*This table reports adjusted hypoglycemia odds ratios (ORs) for sodium thresholds (130–145) vs reference 138.8; lower sodium ranges have strong clinical interpretability, making these ORs highly actionable. With the smallest P (overall) (0.0785) among biomarkers, sodium shows statistically detectable effects at hyponatremia values (130, 135; OR < 1, P~0.018), while 140 has a borderline P-value (~0.0525) and 145 shows high uncertainty (wide CI, P = 0.669). This clearer signal at lower thresholds, along with consistent directionality across adjacent values, makes sodium the most promising candidate for threshold-based interpretation.*

RCS-adjusted logistic regression statistical results for individual biomarkers

| **Biomarker** | **Reference value** | ***P* (overall)** | ***P* (nonlinear)** | **Knots** |
| --- | --- | --- | --- | --- |
| Hemoglobin | 139.0 | 0.360 | 0.890 | [-1.6348, -0.2929, 0.4241, 1.6124] |
| Potassium | 4.05 | 0.173 | 0.964 | [-1.3228, -0.1723, 0.0526, 1.4712] |
| Sodium | 138.8 | 0.0785 | 0.808 | [-1.8229, -0.0491, 0.2117, 1.2552] |

*RCS-adjusted logistic regression results for Hb, K^+^, Na^+^; Na shows strongest association, no biomarkers exhibit significant nonlinearity.*
