# Supplementary material for: Development and validation of an interpretable machine learning model for predicting in-hospital hypoglycemia in adults with type 1 diabetes mellitus: a multicenter retrospective study
Source: Front Endocrinol (Lausanne). 2026 Apr 17;17:1816599. doi: 10.3389/fendo.2026.1816599 (PMC13140310; doi:10.3389/fendo.2026.1816599)
Supplement: Supplementary file 8 [file Table7.docx]

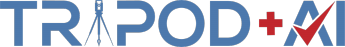


TRIPOD+AI for Abstracts

| **Section and item** | **Checklist item** | **Reported on page** |
| --- | --- | --- |
| **Title** |  |  |
| 1 | Identify the study as developing or evaluating the performance of a multivariable prediction model, the target population, and the outcome to be predicted | Title |
| **Background** |  |  |
| 2 | Provide a brief explanation of the healthcare context and rationale for developing or evaluating the performance of all models | Abstract-Background |
| **Objectives** |  |  |
| 3 | Specify the study objectives, including whether the study describes model development, evaluation, or both | Abstract-Background |
| **Methods** |  |  |
| 4 | Describe the sources of data | Abstract-Methods |
| 5 | Describe the eligibility criteria and setting where the data were collected | Abstract-Methods |
| 6 | Specify the outcome to be predicted by the model, including time horizon of predictions in case of prognostic models | Abstract-Methods |
| 7 | Specify the type of model, a summary of the model-building steps, and the method for internal validation† | Abstract-Methods |
| 8 | Specify the measures used to assess model performance (eg, discrimination, calibration, clinical utility) | Abstract-Methods |
| **Results** |  |  |
| 9 | Report the number of participants and outcome events | Abstract-Results |
| 10 | Summarise the predictors in the final model† | Abstract-Results |
| 11 | Report model performance estimates (with confidence intervals) | Abstract-Results |
| **Discussion** |  |  |
| 12 | Give an overall interpretation of the main results | Abstract-Conclusions |
| **Registration** |  |  |
| 13 | Give the registration number and name of the registry or repository | / |
